# Semi‐Mechanistic Joint Population Pharmacokinetic Modeling of the Novel Antituberculosis Drug Sorfequiline and Its Metabolite M3 in Healthy Volunteers

**DOI:** 10.1002/psp4.70331

**Published:** 2026-09-11

**Authors:** Jose M. Calderin, Jerry Nedelman, David H. Salinger, Thanakorn Vongjarudech, Paolo Denti, Elin M. Svensson

**Affiliations:** ^1^ Division of Clinical Pharmacology, Department of Medicine University of Cape Town Cape Town South Africa; ^2^ Global Alliance for TB Drug Development New York USA; ^3^ Certara, Inc Princeton USA; ^4^ Department of Pharmacy Uppsala University Uppsala Sweden; ^5^ Department of Pharmacy, Pharmacology and Toxicology Radboud Institute of Health Sciences, Radboud University Medical Center Nijmegen the Netherlands

**Keywords:** diarylquinoline, joint parent–metabolite modeling, M3, NONMEM, population pharmacokinetics, semi‐mechanistic modeling, sorfequiline, TBAJ‐876, tuberculosis

## Abstract

Sorfequiline is a novel diarylquinoline in development for treatment of tuberculosis. In vitro data suggest metabolism via CYP3A4, carrying the potential for presystemic metabolism and drug–drug interactions. Its major metabolite, M3, is pharmacologically active. We developed a semi‐mechanistic joint population pharmacokinetic model of sorfequiline and M3 in healthy volunteers. Data were pooled from two studies (CL‐001 and CL‐002). CL‐001 comprised a single‐ascending‐dose part (10–800 mg suspension fasted plus 100 mg fed), a multiple‐ascending‐dose part (25, 75, or 200 mg/day for 14 days, fed), and a relative bioavailability assessment including 100 mg in tablet form under fed and fasted conditions. In CL‐002, fed participants received 200 mg/day suspension for 8 days followed by 165 mg/day for 4 days. Plasma concentrations were quantified using HPLC–MS/MS, and pharmacokinetic modeling and simulations were conducted in NONMEM. A three‐compartment disposition model for both analytes, with transit‐compartment absorption, adequately described the data. A fraction of the dose was assumed to be absorbed as M3 due to gut first‐pass metabolism. This fraction was 46% (95% CI: 41%–50%) in fasted and 15% (13%–19%) in fed participants. Overall bioavailability decreased by 46% (32%–61%) at fasted doses ≥ 400 mg. Model‐based simulations showed that exposures for all investigated regimens remained below safety reference values. Food reduced the fraction of sorfequiline dose absorbed as M3, likely due to limited gut first‐pass metabolism. The developed model provides an initial platform for future pharmacokinetic/pharmacodynamic analyses and simulation of both analytes' exposures under drug–drug interaction scenarios.

## Study Highlights

- What is the current knowledge on the topic?
  ○ Sorfequiline is a novel diarylquinoline in clinical development for tuberculosis treatment. Phase 1 studies showed it was well tolerated, and food affected exposure to sorfequiline and its active metabolite, M3. In vitro data suggest CYP3A4‐mediated metabolism, raising potential for presystemic biotransformation and drug–drug interactions (DDIs).
- What question did this study address?
  ○ This study developed the first joint parent–metabolite population pharmacokinetic model for sorfequiline and M3 and explored the contribution of presystemic metabolism to both analytes' exposure.
- What does this study add to our knowledge?
  ○ The model indicates extensive presystemic gut metabolism of sorfequiline. Food reduced this process, shifting parent–metabolite exposure toward sorfequiline and supporting fed administration. It identified reduced bioavailability at 400 and 800 mg under fasted conditions. Simulations supported the safety of 25‐, 50‐, and 100‐mg doses evaluated in the Phase 2 study (NCT06058299).
- How might this change drug discovery, development, and/or therapeutics?
  ○ This model provides an initial framework to simulate DDI effects on sorfequiline and M3 exposures, support future pharmacokinetic/pharmacodynamic analyses, and inform model‐based dose optimization.

## 1 Introduction

Tuberculosis (TB) remains one of the leading causes of death from infectious diseases worldwide. In 2024, an estimated 10.7 million cases and 1.23 million deaths were reported [1]. Despite the availability of effective regimens for drug‐sensitive TB, incidence has declined by just 29% since 2015, far below the End TB Strategy milestone of a 75% reduction by 2025 [1]. Disease control is hindered by poor adherence to lengthy treatments and the growing prevalence of multidrug‐resistant TB (MDR‐TB) [2], highlighting the urgent need for novel drugs and improved treatment strategies.

The introduction of bedaquiline (BDQ) in 2012, a diarylquinoline that inhibits mycobacterial ATP synthase, improved MDR‐TB treatment success and shortened treatment duration [3]. However, emerging resistance raises concerns about its long‐term effectiveness. A recent study reported a global BDQ resistance prevalence of 5.7%, increasing to 10.4% in South Africa, with acquired resistance of approximately 5.5% in treated cohorts [4]. In another South African cohort with phenotypic BDQ‐resistant TB, TB‐free survival at 18 months was 52%, mortality was 23%, and the median time to sputum negativity was substantially longer than in BDQ‐susceptible controls (175 vs. 32 days) [5].

Sorfequiline (formerly TBAJ‐876), an analogue of BDQ, is a therapeutic candidate under evaluation for both drug‐sensitive and MDR‐TB. It shares BDQ's mechanism of action but shows tenfold lower minimum inhibitory concentrations against *Mycobacterium tuberculosis* , including BDQ‐resistant Rv0678 mutants [6, 7]. Sorfequiline is primarily metabolized by oxidative N‐demethylation, with in vitro data suggesting a key role for CYP3A4 in this pathway. Its major active metabolite, N‐desmethyl‐sorfequiline (M3), is analogous to BDQ's N‐desmethyl metabolite (BDQ‐M2), and exhibits at least tenfold greater bactericidal potency than BDQ‐M2 [8]. Sorfequiline has lower lipophilicity than BDQ, a shorter elimination half‐life after 7 days of dosing in mice (56.8 vs. 112 h, respectively), and the potential for a lower risk of cardiotoxicity due to reduced hERG channel affinity [9, 10]. In murine TB models, sorfequiline was well tolerated, with activity partially attributed to M3 due to its higher exposure compared to the parent compound and strong bactericidal activity [9]. In healthy volunteers, sorfequiline administered once daily for 14 days was also well tolerated, with no clinically significant QTc interval prolongation observed. Noncompartmental pharmacokinetic analysis showed a food effect on the parent–metabolite exposure ratio, with higher sorfequiline exposure and lower M3 exposure in the fed state than under fasted conditions [11].

Given the role of CYP3A4 in sorfequiline metabolism, this drug may undergo extensive presystemic biotransformation because CYP3A4 is highly expressed and active in the intestine and liver [12]. Moreover, as sorfequiline is being evaluated in combination regimens for TB and TB/HIV co‐infection (NCT06058299), it carries a risk of drug–drug interactions (DDIs), since several anti‐TB and antiretroviral drugs are CYP3A4 modulators [13]. While the impact of CYP3A4‐mediated DDIs on parent exposure is relatively straightforward to predict, the consequences for M3 are more complex. Metabolite exposure reflects the balance between its formation from the parent and its own clearance. Enzyme induction can increase parent clearance, potentially increasing metabolite formation, but it may also increase metabolite clearance, resulting in either higher or lower net exposure. Enzyme inhibition produces the opposite interplay, with similarly uncertain effects on metabolite concentrations. Because the efficacy and safety of sorfequiline depend on combined parent–metabolite exposure [9], accurate prediction of both analytes under different dosing regimens and DDI scenarios is essential for regimen design.

In this context, a joint parent–metabolite pharmacokinetic model provides a valuable quantitative tool. Joint modeling of concentration–time data for both analytes can be used to characterize parent and metabolite pharmacokinetics simultaneously, explore presystemic metabolite formation and its contribution to systemic exposure, and assess variability and covariate effects on both analytes within a unified framework [14]. In this study, we aimed to develop a semi‐mechanistic joint population pharmacokinetic model of sorfequiline and its metabolite M3.

## 2 Methods

### 2.1 Study Design

This pharmacokinetic analysis pooled data from two Phase 1 studies (CL‐001 and CL‐002) conducted in healthy volunteers. Ethics approval for CL‐001 and CL‐002 was obtained from IntegReview Institutional Review Board (Austin, Texas, USA) and Advarra (Columbia, Maryland, USA), respectively. Study CL‐001 (NCT04493671) was a three‐part, partially blind, placebo‐controlled trial conducted in San Antonio, Texas, United States, that evaluated the safety, tolerability, and pharmacokinetics of sorfequiline. Study CL‐002 (NCT05526911) was an open‐label DDIs study conducted in Fair Lawn, New Jersey, United States, that evaluated the safety, tolerability, and the induction potential of sorfequiline on CYP3A4 and P‐glycoprotein (P‐gp), as well as its inhibition potential on P‐gp.

### 2.2 Study CL‐001

Study CL‐001 consisted of three parts: A combined single ascending dose (SAD) study with a food‐effect cohort, a multiple ascending dose (MAD) study, and a relative bioavailability (RBA) study. In the SAD part, participants were randomized into treatment groups to receive a single dose of sorfequiline oral suspension at 10, 25, 50, 100, 200, 400, or 800 mg under fasted conditions, with an additional food‐effect group receiving 100 mg under fed conditions. In the MAD part, participants were randomized into one of three treatment groups to receive a once‐daily dose of sorfequiline oral suspension at 25, 75, or 200 mg under fed conditions for 14 days. In the RBA part, participants were randomized into three treatment groups to receive either a single 100 mg tablet under fasted conditions, a single 100 mg tablet under fed conditions, or four 25 mg tablets under fasted conditions.

### 2.3 Study CL‐002

This study evaluated the effects of sorfequiline on CYP3A4 and P‐gp, using midazolam and digoxin as probe substrates. Participants received 2 mg midazolam oral syrup on days 1 and 20, and a 0.25 mg digoxin tablet on days 2 and 21. Beginning on day 6, sorfequiline was administered once daily as an oral suspension under fed conditions: 200 mg from days 6 to 13, followed by 165 mg from days 14 to 19. On days 20 and 21, participants received a single 200 mg suspension dose under fasted conditions; on days 22 to 24, they received 150 mg as an oral suspension under fed conditions. Probe substrate data were not included in our analysis.

### 2.4 Pharmacokinetic Sampling

In all parts of study CL‐001, blood samples were collected at 0.5, 1, 2, 3, 4, 5, 6, 8, 10, 12, 16, 20, and 24 h post‐dose. In the SAD and RBA parts, additional samples were collected at 28, 32, 36, 40, 48, 54, 60, 66, 72, 80, 88, 96, 120, 144, and 168 h post‐dose, and in some SAD cohorts, sparse sampling was extended to later time points up to 70 days post‐dose. In the MAD part, intensive sampling was performed on day 1 and day 14, with additional pre‐dose samples collected daily between these visits, and further sparse sampling continued beyond day 14 at various time points through day 139.

In study CL‐002, blood samples for sorfequiline analysis were collected on day 17 (12 days after initiation of sorfequiline dosing) at pre‐dose and at 0.5, 3, 4, 5, 6, 7, 8, 12, 16, 20, and 24 h post‐dose.

Samples were processed immediately after collection and stored at −80°C (±10°C) until analysis. Concentrations of sorfequiline and M3 were measured from the same sample at Alliance Pharma Inc. using validated high‐performance liquid chromatography–tandem mass spectrometry assays. The lower limit of quantification (LLOQ) was 1 ng/mL for both analytes [11]. Participants' characteristics, including weight, height, sex, race, ethnicity, and serum chemistry, were collected at enrolment.

### 2.5 Pharmacokinetic Modeling

Nonlinear mixed‐effects modeling was conducted using NONMEM version 7.5.1 to develop a joint population pharmacokinetic model describing the concentration–time data of both sorfequiline and M3. The first‐order conditional estimation with eta–epsilon interaction algorithm was applied throughout the modeling process [15]. Model development followed a sequential approach: It began with concentrations of the parent drug from the SAD cohort; then the population parameters were fixed, and M3 data from the same cohort were incorporated; finally, all population parameters were unfixed, and parent and metabolite parameters were estimated simultaneously. Once a suitable model was established, data from the remaining cohorts were sequentially added to the analysis.

One‐, two‐, and three‐compartment disposition models with first‐order elimination were evaluated for both analytes. For sorfequiline, first‐order absorption was tested with or without lag‐time or transit compartments to account for absorption delay. Complete conversion of sorfequiline to M3 was assumed, and a correction factor was incorporated into the model to account for the molecular weight difference between sorfequiline (657.55 g/mol) and M3 (643.55 g/mol). A parameter describing the fraction of dose absorbed as metabolite (FAM) due to presystemic metabolism in the gut was tested in the model.

The effect of body size on disposition parameters was tested using allometric scaling based on total body weight (WT) or fat‐free mass (FFM) estimated with the formula by Janmahasatian et al. [16]. The allometric exponents for clearances and volumes were fixed to 0.75 and 1, respectively [17]. The effects of other covariates (including fed vs. fasted status, formulation, number of tablets, dose, race, and ethnicity) were tested.

The likelihood ratio test using the difference in objective function value (OFV) was used to compare nested models, with ΔOFV assumed to follow a χ² distribution with n degrees of freedom (df), where n is the number of additional estimated parameters. A supervised stepwise approach was applied for model development: A *p*‐value of 0.05 (ΔOFV ≥ 3.84, df = 1) was used for inclusion of an additional parameter, while a *p*‐value of 0.01 (ΔOFV ≥ 6.63, df = 1) was required for its retention during backward elimination. For non‐nested models, the Akaike information criterion (AIC) was used for model comparison. Beyond statistical significance, model development decisions were informed by diagnostic plots (e.g., visual predictive checks [VPCs]), as well as physiological plausibility and clinical relevance.

Random effects were included on pharmacokinetic parameters if statistically significant, assuming a log‐normal distribution [15], except for FAM (fraction of dose absorbed as metabolite), which was modeled using a logit transformation [15]. Between‐subject variability (BSV) was evaluated for disposition parameters, and between‐occasion variability (BOV) for absorption parameters, with an occasion defined as a dosing event and its subsequent observations.

Residual unexplained variability (RUV) was described using a combined additive and proportional error model for each analyte, with the additive component constrained to be at least 20% of the LLOQ. Because sorfequiline and M3 were quantified simultaneously from the same samples, a correlation coefficient between their RUV terms was tested using the L2 method [18]. Concentrations below the limit of quantification (BLQ) were handled using the M7+ method proposed by Wijk et al. [19]: BLQ values were imputed as zero, and the additive component of their RUV was increased by 100% of the LLOQ. The precision of parameter estimates in the final model, expressed as 95% confidence intervals (CI), was evaluated using the sampling importance resampling (SIR) method after confirming adequate SIR convergence [20].

### 2.6 Simulations

The final model was used to simulate sorfequiline and M3 exposures over 12weeks across different dosing regimens, assuming the same dosing conditions as an ongoing Phase 2 trial (NCT06058299) (oral tablet administration under fed conditions) and the same maintenance doses (25, 50, and 100 mg/day). In addition, regimens incorporating a 2‐week loading phase followed by 10 weeks of maintenance dosing were evaluated. Loading doses were set at 2‐, 3‐, and 4‐fold the corresponding maintenance dose. The CL‐002‐specific effect on relative bioavailability was not applied in the simulations because its source and relevance beyond CL‐002 could not be established.

Simulated sorfequiline and M3 exposures were reported as the sum of their daily AUC_0–24h_ values (AUC_0–24h,sum_ = AUC_0–24h,sorfequiline_ + AUC_0–24h,M3_) and compared with safety reference values. Two exposure‐based safety thresholds from dog toxicity studies in which cardiotoxicity was reported were used to assess whether the simulated regimens were within safety margins: An AUC_0–24h,sum_ of 62.2 mg·h/L, corresponding to the no‐observed‐adverse‐effect level (NOAEL), and an AUC_0–24h,sum_ of 127.5 mg·h/L, corresponding to the lowest‐observed‐adverse‐effect level (LOAEL) [11] (the NOAEL value of 27.2 mg·h/L reported in this reference was later readjudicated; the currently accepted value is the one used here, based on TB Alliance internal data). Daily AUC_0–24h_ values were summed in mass units (mg·h/L), consistent with the safety reference values. Because sorfequiline and M3 have similar molecular weights (657.55 vs. 643.55 g/mol; ~2.1% difference), the mass‐based sum closely approximates a molar‐based sum.

## 3 Results

### 3.1 Study Data

A total of 4638 samples were obtained from 138 participants across studies CL‐001 and CL‐002. BLQ data accounted for 4.0% (179/4,638) of sorfequiline and 17.0% (784/4,638) of M3, mostly at low doses, as expected. Metabolite data from the 10 mg dose group in the SAD part of study CL‐001 were excluded from the analysis, as all samples were BLQ. Exploratory plots of the observed sorfequiline and M3 concentration‐time data are provided in the (Figure S1–S9). Demographic characteristics of study participants are summarized in Table 1.

**TABLE 1:** Demographic characteristics of study participants.

*N*	CL‐001 study			CL‐002 study	Overall
	SAD cohort	MAD cohort	RBA cohort		
	55	27	30	26	138
Female	22 (40)	14 (52)	23 (77)	13 (50)	72 (52)
Age (years)	34 (19–50)	35 (19–48)	36 (19–50)	34 (20–55)	35 (19–55)
Weight (kg)	76 (52–100)	77 (56–115)	78 (57–94)	76 (49–153)	77 (49–153)
Height (cm)	166 (148–187)	167 (154–198)	174 (152–192)	170 (149–185)	169 (148–198)
FFM (kg)^a^	44 (34–72)	49 (37–82)	60 (36–69)	54 (33–70)	53 (33–82)
Race					
White	36 (65)	20 (74)	16 (54)	7 (27)	79 (57)
American Indian or Alaska Native	1 (2)	0 (0)	0 (0)	0 (0)	1 (< 1)
Asian	0 (0)	1 (4)	1 (3)	0 (0)	2 (1)
Black or African American	15 (27)	6 (22)	12 (40)	19 (73)	52 (38)
Multiple	3 (6)	0 (0)	1 (3)	0 (0)	4 (3)
Ethnicity					
Hispanic or Latino	34 (62)	13 (48)	15 (50)	8 (31)	70 (51)
Not Hispanic or Latino	21 (38)	14 (52)	15 (50)	18 (69)	68 (49)

*Note:* Data are presented as median (min–max) or *n* (%).

^a^ Fat‐free mass estimated with the formula by Janmahasatian et al. [16].

### 3.2 Pharmacokinetic Modeling

**FIGURE 1:**
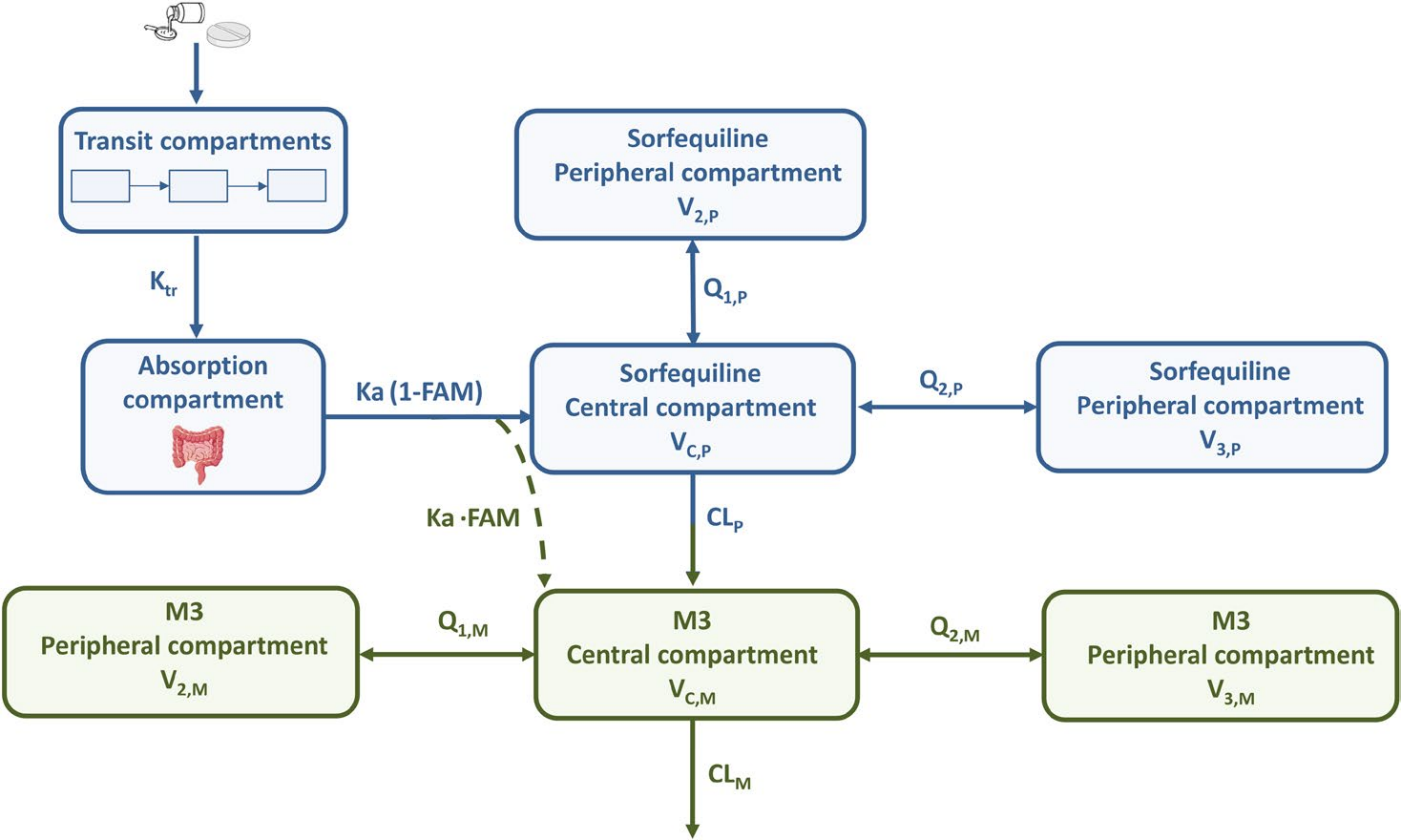
Schematic representation of the final model. Ktr: Transit rate constant; Ka: Absorption rate constant; FAM: Fraction of dose absorbed as metabolite due to gut presystemic metabolism; CL_P_: Clearance of the parent; CL_M_: Clearance of the metabolite; Q: Intercompartmental clearance; V: Volume of distribution.

The pharmacokinetics of sorfequiline and M3 were described by a structural model consisting of three compartments for each analyte, with first‐order absorption and elimination, and using the clearance of the parent as input for the metabolite. A schematic representation of the final model is depicted in Figure 1. Implementing absorption delay using 3 transit compartments significantly improved model fit (ΔOFV = −26.1, df = 2, *p* < 0.001) compared with no delay and provided a better fit than a lag‐time model (ΔOFV = −19.5, df = 1, *p* < 0.001). Allometric scaling with FFM (ΔOFV = −11.6) better described the influence of body size on the disposition parameters of sorfequiline and M3 compared with WT (ΔOFV = −5.8).

Significant correlations were identified between the BSV in clearance of sorfequiline and M3 (71.3%; 95% CI: 54.8%–90.8%), as well as between their RUV (78.6%; 95% CI: 77.2%–80.1%). Implementation of the FAM parameter, describing the fraction of dose absorbed as M3 due to presystemic metabolism in the gut, improved model fit (ΔOFV = −38.4, df = 1, *p* < 0.001) and yielded an estimated value of 46.4% (95% CI: 41.7%–50.0%) under fasted conditions.

The model identified a significant effect of food on FAM (ΔOFV = −14.1, df = 1, *p* < 0.001), reducing its estimated value to 15.7% (95% CI: 13.4%–19.0%) in fed individuals. Food intake was also associated with a faster absorption rate (ΔOFV = −16.5; df = 1; *p* < 0.001), with an estimated increase of 13.6% (95% CI: 8.9%–18.5%) in this parameter. In addition, a significant food effect on the mean transit time of the tablet formulation was observed (ΔOFV = −37.5; df = 1; *p* < 0.001), with an estimated increase of 188% (95% CI: 116%–250%).

A 46.7% (95% CI: −60.6%–32.3%) decrease in bioavailability (ΔOFV = −19.6, df = 1, *p* < 0.001) was estimated for both the 400‐ and 800‐mg doses. No significant effects of tablet number (4 × 25 mg vs. 1 × 100 mg) or any other evaluated covariates were identified on pharmacokinetic parameters.

The model identified significantly higher bioavailability of the suspension formulation in study CL‐002 (ΔOFV = −71.6; df = 1; *p* < 0.001), with an estimated 47.2% (95% CI: 36.2%–61.2%) increase relative to study CL‐001. Although the distribution of some demographic characteristics differed between CL‐001 and CL‐002 (Table 1), covariate analysis (conducted before and after inclusion of the study effect) did not identify any measured covariate that explained the observed difference in bioavailability.

VPCs for the final model are provided in the (Figures S10–S17) and show good agreement between observations and model predictions. The NONMEM control stream for the final model is also provided in the (Section S1). The final pharmacokinetic parameter estimates with their respective 95% CI are presented in Table 2.

**TABLE 2:** Final parameter estimates and 95% confidence intervals.

Parameter (units)	Typical value (95% CI)^a^	Parameter variability, %CV (95% CI)^a^
Sorfequiline		
Clearance, CL_P_ (L/h)^b^, ^g^	4.24 (3.74–4.66)	BSV: 53.8 (48.0–61.5)
Central volume of distribution, V_C,*p*_ (L)^b^, ^g^	17.1 (14.58–19.27)	
Intercompartmental clearance, Q_1,*p*_ (L/h)^b^, ^g^	6.64 (6.19–7.07)	
Peripheral volume of distribution, V_2,*p*_ (L)^b^, ^g^	214 (199–227)	
Intercompartmental clearance, Q_2,*p*_ (L/h)^b^, ^g^	10.4 (9.96–10.7)	
Peripheral volume of distribution, V_3,*p*_ (L)^b^, ^g^	9180 (8717–9672)	
First‐order absorption rate constant, Ka (h^−1^)	0.127 (0.120–0.133)	BOV: 26.3 (23.3–29.7)
Bioavailability, F (.)	1 Fixed	BOV: 53.1 (48.1–60.5)
Mean absorption transit time, MTT (h)	1.13 (1.02–1.24)	BOV: 48.6 (43.1–55.7)
Number of absorption transit compartments, NN (.)^d^	3 Fixed	
M3		
Metabolite clearance, CL_M_ (L/h)^b^, ^g^	38.4 (35.0–41.2)	BSV: 38.3 (33.3–42.5)
Central volume of distribution, V_C,M_ (L)^b^, ^g^	3330 (3153–3566)	
Intercompartmental clearance, Q_1,M_ (L/h)^b^, ^g^	43.5 (39.0–49.1)	
Peripheral volume of distribution, V_2,M_ (L)^b^, ^g^	6460 (6036–7148)	
Intercompartmental clearance, Q_2,M_ (L/h)^b^, ^g^	192 (177–213)	
Peripheral volume of distribution, V_3,M_ (L)^b^, ^g^	2870 (2596–3191)	
Fraction of dose absorbed as metabolite, FAM (.)^f^	0.464 (0.417–0.50)	BSV: 31.8 (27.5–35.5)
Covariates		
Food effect on Ka (%)	+13.6 (+8.99 − +18.5)	
Food effect on tablet MTT (%)	+188 (+116 − +250)	
Change in F in Study CL‐002 (%)	+47.2 (+36.2 − +61.2)	
Change in F at doses ≥ 400 mg (%)	−46.7 (−60.6 – −32.3)	
FAM in fed individuals (.)	0.157 (0.134–0.190)	
Residual unexplained variability		
Sorfequiline proportional error (%)	21.7 (21.2–22.2)	
Sorfequiline additive error (mg/L)^c^	2 × 10^−4^ Fixed	
M3 proportional error (%)	16.5 (16.0–17.0)	
M3 additive error (mg/L)^c^	2 × 10^−4^ Fixed	
Correlations		
BSVCL correlation (Sorfequiline vs. M3) (%)	71.3 (54.8–90.8)	
RUV correlation (Sorfequiline vs. M3) (%)^e^	78.6 (77.2–80.1)	

Abbreviations: BOV, between‐occasion variability; BSV, between‐subject variability; CI, confidence interval; CV, coefficient of variation; RUV, residual unexplained variability.

^a^ Values in parentheses represent the 95% confidence interval, computed using sampling importance resampling (SIR) on the final model.

^b^ All the disposition parameters were allometrically scaled with a fixed coefficient of 0.75 for clearances and 1 for volumes. The reported values refer to the typical individual in the cohort with a fat‐free mass of 53 kg.

^c^ The estimate of the additive component of the residual unexplained variability did not significantly differ from its lower boundary of 20% of LLOQ; it was consequently fixed to this value.

^d^ The number of transit compartments was fixed to 3 (based on the model's estimation) to enhance stability. A sensitivity analysis showed the value of the number of transit compartments was not critical for overall model fit.

^e^ The correlation between the residual variability models for sorfequiline and M3 was implemented using the NONMEM Level 2 (L2) data item.

^f^ BSV for FAM was modeled on the logit scale (ω^2^ = 0.429); the reported CV% was obtained by simulation‐based back‐transformation to the original scale.

^g^ Disposition parameters for sorfequiline were estimated relative to the true oral bioavailability (F). Similarly, disposition parameters for M3 were estimated relative to the true fraction of sorfequiline metabolized to M3.

### 3.3 Simulations

**FIGURE 2:**
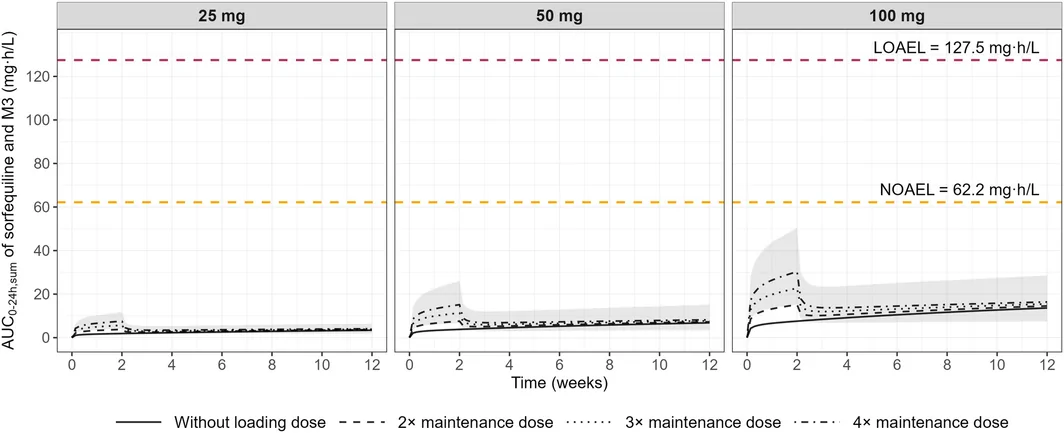
Model‐predicted summed exposure (AUC_0–24h,sum_ = AUC_0–24h,sorfequiline_ + AUC_0–24h,M3_) over 12 weeks of once‐daily dosing, stratified by maintenance dose (25, 50, and 100 mg). Lines represent the median predicted profiles across 500 simulation replicates of the typical individual in the study population (FFM: 53 kg) under each dosing regimen. Line type denotes the loading strategy: No loading dose, or a loading dose equal to 2‐, 3‐, or 4‐fold the maintenance dose during the first 2 weeks. Shaded areas represent the 90% model‐predicted interval for the regimens with a loading dose equal to 4‐fold the maintenance dose during the first 2 weeks. Horizontal dashed lines indicate preclinical safety reference thresholds from dog toxicity studies in which cardiotoxicity was reported.

Model‐predicted AUC_0–24h,sum_ profiles over 12 weeks of once‐daily dosing are shown in Figure 2. Across all simulated regimens (with or without loading doses), typical predicted AUC_0–24h,sum_ remained well below the preclinical safety thresholds. Steady state was not reached in any regimen by week 12. Loading doses increased early exposure; after discontinuation of the loading phase, predicted AUC_0–24h,sum_ decreased, and the profiles converged, with exposure after week 4 largely determined by the maintenance dose.

## 4 Discussion

We developed a population pharmacokinetic model that jointly characterizes sorfequiline, a novel antimycobacterial drug, and its major active metabolite M3, using data from two studies in healthy volunteers. The model semi‐mechanistically describes presystemic M3 formation via intestinal metabolism of sorfequiline. Food shifted the parent–metabolite exposure balance toward sorfequiline by reducing the fraction of the dose converted presystemically to M3. The model also suggested reduced bioavailability at higher doses, likely due to limited solubility under fasted conditions. Finally, model‐based simulations indicated that summed sorfequiline and M3 exposures across all evaluated dosing regimens remained below established preclinical safety thresholds, providing preliminary support for the ongoing clinical evaluation of sorfequiline.

In murine TB models, M3 exposures exceeded those of sorfequiline after 4 weeks of oral treatment [9]. Our model estimated that, under fasted conditions, 46% of the absorbed fraction of the sorfequiline dose contributed to systemic M3 input. This does not imply similar sorfequiline and M3 concentrations because the parent–metabolite exposure ratio also depends on their respective apparent clearances and volumes of distribution, as well as on sorfequiline elimination pathways other than M3 formation. Given the role of CYP3A4 in sorfequiline metabolism, and evidence that intestinal CYP3A4 may contribute more to the presystemic metabolism of CYP3A4 substrates than hepatic CYP3A4 [12], these findings are consistent with substantial first‐pass metabolism of sorfequiline in the gut. Providing indirect support for this hypothesis, the model estimated that only 15% of the absorbed dose was present as M3 under fed conditions. Sorfequiline is a lipophilic compound (cLogP≈5.15) [7]. Following a high‐calorie, high‐fat meal, bile salt secretion and mixed micelle formation increase, improving luminal solubilization and dissolution [21]. Within enterocytes, the concomitant rise in chylomicron production facilitates lymphatic transport of lipophilic drugs, thereby partially bypassing enterocyte CYP3A4 metabolism [21, 22]. At the same time, greater intestinal permeability and enhanced lymphatic uptake reduce enterocyte residence time, limiting the opportunity for CYP3A4 extraction [23]. Although gastric emptying is delayed under fed conditions, once the drug reaches the small intestine, the presence of bile salts, fluid secretion, and increased mucosal perfusion steepen the concentration gradient across the epithelium, accelerating absorption compared with fasting conditions [24], as observed in our analysis.

A delay in the appearance of sorfequiline concentrations was observed under fed conditions following tablet administration. This may be attributable to slower gastric emptying and increased viscosity of gastric contents in the fed state, which can prolong tablet residence in the stomach and delay delivery of the drug to the intestinal absorption site [23, 25]. In addition, food components, particularly fat, may form hydrophobic layers around the tablet surface, reducing water penetration and thereby delaying disintegration and dissolution [26].

Reduced bioavailability was observed when higher doses of 400 and 800 mg were administered under fasted conditions, likely due to solubility‐limited absorption. At higher doses, the drug amount can exceed the dissolution capacity of gastrointestinal fluids, particularly for lipophilic compounds in the fasted state, resulting in incomplete dissolution and a reduced fraction of drug available for absorption [27]. However, these doses are above the dose range (25, 50, and 100 mg) currently being evaluated in the Phase 2 clinical trial (NCT06058299).

A 47% higher bioavailability was estimated in CL‐002 than in CL‐001; however, the reasons for this difference remain unclear. An effect of the co‐administered probe drugs used in CL‐002, as part of the DDI assessment, on sorfequiline pharmacokinetics is unlikely. Meals were also standardized across studies: Participants received a high‐calorie, high‐fat breakfast consumed within 30 min before dosing. In addition, although race and ethnicity distributions differed (CL‐002 included more Black or African American participants (73% vs. 29%) and more non‐Hispanic participants (69% vs. 45%) than CL‐001), these covariates did not explain the observed difference, and other baseline demographics were broadly comparable.

As the individual contributions of sorfequiline and M3 to toxicity are not fully elucidated, we evaluated safety assuming equal contributions from both analytes. Simulations indicated that summed sorfequiline and M3 exposure remained within the safety margin across the evaluated regimens. Given sorfequiline's long elimination half‐life and gradual accumulation, none of the simulated regimens reached steady state by week 12. Simulations targeted a shorter treatment course than the 24 weeks recommended for bedaquiline, as preclinical findings suggest greater sterilizing activity for sorfequiline‐containing regimens than for bedaquiline‐containing regimens, supporting the potential for treatment shortening [10]. Our simulations of regimens incorporating a loading‐dose phase may inform future regimen design, given that higher early exposures may enhance early bactericidal activity, as has been observed with bedaquiline [28].

Although our pharmacokinetic analysis indicated reduced bioavailability at doses ≥ 400 mg for the oral suspension under fasted conditions, we did not include this effect in the simulations because its relevance and magnitude for the tablet formulation administered with food are uncertain. By omitting this effect, we simulated higher exposures and therefore provided a conservative (worst‐case) scenario for safety evaluation. Consistent with our model‐based safety assessments, a recent report from the ongoing Phase 2 trial indicated that after 8 weeks of once‐daily dosing (25, 50, and 100 mg), sorfequiline was well tolerated, with comparable safety across dose levels and fewer grade ≥ 3 treatment‐emergent adverse events than in the standard TB treatment arm [29].

Our analysis has several limitations. The model was developed in healthy volunteers, which may limit extrapolation to patients with TB because differences in protein binding and body composition, among other factors, may affect drug disposition. Model predictions for this population should therefore be considered exploratory until the model is evaluated and, if necessary, updated using patient pharmacokinetic data. Intravenous data for sorfequiline were not available, which limited the ability to disentangle absolute oral bioavailability from presystemic metabolism. Parent disposition parameters were estimated relative to bioavailability, while metabolite disposition parameters were estimated relative to the unknown true fraction of sorfequiline converted to M3. In addition, the absence of pharmacokinetic data at 400‐ and 800‐mg doses under fed conditions limited the evaluation of food effects across the full dose range; nevertheless, these doses are above those evaluated in the Phase 2 trial (25, 50, and 100 mg). Finally, translation of dog‐derived safety reference values to humans carries some uncertainty, as the proportion of M3 formed appears higher in dogs than in humans (TB Alliance internal data); however, safety data from ongoing studies to date are reassuring [29].

In summary, we developed a joint parent–metabolite population pharmacokinetic model that semi‐mechanistically describes the intestinal first‐pass metabolism of sorfequiline. Our findings support the rationale for administering sorfequiline with food, as food increased sorfequiline exposure and reduced presystemic M3 formation, likely by limiting intestinal first‐pass metabolism. The model also indicates dose‐dependent bioavailability at higher doses, at least under fasting conditions, providing information relevant to future dose‐selection analyses. Additionally, model‐based simulations provide preliminary support for the continued clinical evaluation of the dose levels in the ongoing Phase 2 trial. The developed model provides a semi‐mechanistic platform to simulate DDI effects on presystemic and/or systemic conversion processes and their potential impact on sorfequiline and M3 exposures. It can also support pharmacokinetic/pharmacodynamic analyses, inform future clinical study design, and guide the evaluation of sorfequiline dosing regimens.

## Author Contributions

J.M.C., J.N., P.D., and E.M.S. wrote the manuscript; J.N., P.D., and E.M.S. designed the research; J.M.C., J.N., D.H.S., T.V., P.D., and E.M.S. performed the research; J.M.C., J.N., D.H.S., T.V., P.D., and E.M.S. analyzed the data.

## Funding

The modelling analysis was partly supported by a research grant from the Global Alliance for TB Drug Development, New York, USA.

## Conflicts of Interest

Jerry Nedelman is an employee of TB Alliance, which developed the drug studied in this work. All other authors declare no competing interests for this work. As an Associate Editor for *CPT: Pharmacometrics & Systems Pharmacology*, Paolo Denti was not involved in the review or decision process for this paper.

## Supporting information


**Figure S1:** Individual observed concentration‐time profiles of sorfequiline and M3, stratified by dose and food status, during the first 7 days after dosing in the single ascending dose (SAD) cohort of study CL‐001.
**Figure S2:** Individual observed concentration‐time profiles of sorfequiline and M3, stratified by dose, during the first 15 days after the first dose in the multiple ascending dose (MAD) cohort of study CL‐001.
**Figure S3:** Individual observed concentration‐time profiles of sorfequiline and M3, stratified by food status, during the first 7 days after 100 mg tablet administration in the relative bioavailability (RBA) cohort of study CL‐001.
**Figure S4:** Dose‐normalized individual observed concentration‐time profiles of sorfequiline and M3, stratified by dose and food status, during the first 7 days after dosing in the single ascending dose cohort of study CL‐001.
**Figure S5:** Individual observed concentration‐time profiles of sorfequiline and M3 during the first 7 days after 100 mg administration in study CL‐001, stratified by formulation and food status.
**Figure S6:** Individual observed concentration‐time profiles of sorfequiline and M3 on day 12 after sorfequiline initiation in study CL‐002.
**Figure S7:** Individual observed concentration‐time profiles of sorfequiline and M3 over the full sampling period in the single ascending dose cohort of study CL‐001, stratified by dose and food status.
**Figure S8:** Individual observed concentration‐time profiles of sorfequiline and M3 over the full sampling period in the multiple ascending dose cohort of study CL‐001, stratified by dose.
**Figure S9:** Individual observed concentration‐time profiles of sorfequiline and M3 over the full sampling period following 100 mg tablet administration in the relative bioavailability (RBA) cohort of study CL‐001, stratified by food status.
**Figure S10:** Visual predictive check of sorfequiline and M3 concentrations vs. time after dose for the SAD part of the CL‐001 study, stratified by dose level, food status, and analyte.
**Figure S11:** Visual predictive check of sorfequiline and M3 concentrations vs. time after dose for the MAD part of the CL‐001 study, stratified by dose and analyte; all participants were fed.
**Figure S12:** Visual predictive check of sorfequiline and M3 concentrations vs. time after dose for the RBA part of the CL‐001 study, stratified by food status and analyte; all received 100 mg.
**Figure S13:** Visual predictive check of sorfequiline and M3 concentrations vs. time after dose for the CL‐002 study, stratified by analyte.
**Figure S14:** Prediction‐corrected visual predictive check of sorfequiline and M3 concentrations vs. time since first dose for the single‐dose data (SAD and RBA cohorts), stratified by analyte, up to 360 h after dosing.
**Figure S15:** Prediction‐corrected visual predictive check of sorfequiline and M3 concentrations vs. time since first dose for the multiple‐dose data (MAD and CL‐002 cohorts), stratified by analyte, up to 360 h after dosing.
**Figure S16:** Prediction‐corrected visual predictive check of sorfequiline and M3 concentrations vs. time since first dose for the single‐dose data (SAD and RBA cohorts), stratified by analyte.
**Figure S17:** Prediction‐corrected visual predictive check of sorfequiline and M3 concentrations vs. time since first dose for the multiple‐dose data (MAD and CL‐002 cohorts), stratified by analyte.
**Section S1:** NONMEM control stream for the final model.
