# Supplementary material for: Semi‐Mechanistic Joint Population Pharmacokinetic Modeling of the Novel Antituberculosis Drug Sorfequiline and Its Metabolite M3 in Healthy Volunteers
Source: CPT Pharmacometrics Syst Pharmacol. 2026 Sep 11;15(9):e70331. doi: 10.1002/psp4.70331 (PMC13563343; doi:10.1002/psp4.70331)
Supplement: Supplementary file 1 [file PSP4-15-e70331-s001.docx]

**SUPPLEMENTARY MATERIALS**

**Title:** Semi-mechanistic joint population pharmacokinetic modelling of the novel antituberculosis drug sorfequiline and its metabolite M3 in healthy volunteers

**Authors:** Jose M Calderin, Jerry Nedelman^#^, David H Salinger, Thanakorn Vongjarudech, Paolo Denti, Elin M Svensson

**# Corresponding author:** Jerry Nedelman, [jerry.nedelman@tballiance.org](mailto:jerry.nedelman@tballiance.org)

Contents

[**1.**	**Exploratory plots of observed sorfequiline and M3 concentration-time data**	4](#_Toc236907431)

[**Figure S1.** Individual observed concentration-time profiles of sorfequiline and M3, stratified by dose and food status, during the first 7 days after dosing in the single ascending dose (SAD) cohort of study CL-001.	4](#_Toc236907432)

[**Figure S2.** Individual observed concentration-time profiles of sorfequiline and M3, stratified by dose, during the first 15 days after the first dose in the multiple ascending dose (MAD) cohort of study CL-001	5](#_Toc236907433)

[**Figure S3.** Individual observed concentration-time profiles of sorfequiline and M3, stratified by food status, during the first 7 days after 100 mg tablet administration in the relative bioavailability (RBA) cohort of study CL-001.	5](#_Toc236907434)

[**Figure S4.** Dose-normalised individual observed concentration-time profiles of sorfequiline and M3, stratified by dose and food status, during the first 7 days after dosing in the single ascending dose cohort of study CL-001.	6](#_Toc236907435)

[**Figure S5.** Individual observed concentration-time profiles of sorfequiline and M3 during the first 7 days after 100 mg administration in study CL-001, stratified by formulation and food status.	7](#_Toc236907436)

[**Figure S6.** Individual observed concentration-time profiles of sorfequiline and M3 on day 12 after sorfequiline initiation in study CL-002.	7](#_Toc236907437)

[**Figure S7.** Individual observed concentration-time profiles of sorfequiline and M3 over the full sampling period in the single ascending dose cohort of study CL-001, stratified by dose and food status.	8](#_Toc236907438)

[**Figure S8.** Individual observed concentration-time profiles of sorfequiline and M3 over the full sampling period in the multiple ascending dose cohort of study CL-001, stratified by dose.	9](#_Toc236907439)

[**Figure S9.** Individual observed concentration-time profiles of sorfequiline and M3 over the full sampling period following 100 mg tablet administration in the relative bioavailability (RBA) cohort of study CL-001, stratified by food status.	9](#_Toc236907440)

[**2.**	**Visual Predictive Checks (VPCs)**	10](#_Toc236907441)

[**Figure S10.** Visual predictive check of sorfequiline and M3 concentrations versus time after dose for the SAD part of the CL-001 study, stratified by dose level, food status and analyte.	10](#_Toc236907442)

[**Figure S11.** Visual predictive check of sorfequiline and M3 concentrations versus time after dose for the MAD part of the CL-001 study, stratified by dose and analyte; all participants were fed.	11](#_Toc236907443)

[**Figure S12.** Visual predictive check of sorfequiline and M3 concentrations versus time after dose for the RBA part of the CL-001 study, stratified by food status and analyte; all received 100 mg.	12](#_Toc236907444)

[**Figure S13.** Visual predictive check of sorfequiline and M3 concentrations versus time after dose for the CL-002 study, stratified by analyte.	12](#_Toc236907445)

[**Figure S14.** Prediction-corrected visual predictive check of sorfequiline and M3 concentrations versus time since first dose for the single-dose data (SAD and RBA cohorts), stratified by analyte, up to 360 hours after dosing.	13](#_Toc236907446)

[**Figure S15.** Prediction-corrected visual predictive check of sorfequiline and M3 concentrations versus time since first dose for the multiple-dose data (MAD and CL-002 cohorts), stratified by analyte, up to 360 hours after dosing.	14](#_Toc236907447)

[**Figure S16.** Prediction-corrected visual predictive check of sorfequiline and M3 concentrations versus time since first dose for the single-dose data (SAD and RBA cohorts), stratified by analyte.	15](#_Toc236907448)

[**Figure S17.** Prediction-corrected visual predictive check of sorfequiline and M3 concentrations versus time since first dose for the multiple-dose data (MAD and CL-002 cohorts), stratified by analyte.	16](#_Toc236907449)

[**3.**	**S1. NONMEM control stream for the final model**	17](#_Toc236907450)

# 1. **Exploratory plots of observed sorfequiline and M3 concentration-time data**

**Figure S1.** Individual observed concentration-time profiles of sorfequiline and M3, stratified by dose and food status, during the first 7 days after dosing in the single ascending dose (SAD) cohort of study CL-001. M3 data from the 10 mg dose group were excluded from the analysis because most observations were below the assay lower limit of quantification.

**Figure S2.** Individual observed concentration-time profiles of sorfequiline and M3, stratified by dose, during the first 15 days after the first dose in the multiple ascending dose (MAD) cohort of study CL-001.

## **Figure S3.** Individual observed concentration-time profiles of sorfequiline and M3, stratified by food status, during the first 7 days after 100 mg tablet administration in the relative bioavailability (RBA) cohort of study CL-001.

**Figure S4.** Dose-normalised individual observed concentration-time profiles of sorfequiline and M3, stratified by dose and food status, during the first 7 days after dosing in the single ascending dose cohort of study CL-001. M3 data from the 10 mg dose group were excluded from the analysis because most observations were below the assay lower limit of quantification.

**Figure S5.** Individual observed concentration-time profiles of sorfequiline and M3 during the first 7 days after 100 mg administration in study CL-001, stratified by formulation and food status. Oral suspension data are from the SAD cohort, and tablet data are from the RBA cohort.

**Figure S6.** Individual observed concentration-time profiles of sorfequiline and M3 on day 12 after sorfequiline initiation in study CL-002. Participants received 200 mg once daily for 8 days, followed by 165 mg once daily for 4 days, under fed conditions. Time is shown relative to the day 12 dose.

**Figure S7.** Individual observed concentration-time profiles of sorfequiline and M3 over the full sampling period in the single ascending dose cohort of study CL-001, stratified by dose and food status. M3 data from the 10 mg dose group were excluded from the analysis because most observations were below the assay lower limit of quantification.

**Figure S8.** Individual observed concentration-time profiles of sorfequiline and M3 over the full sampling period in the multiple ascending dose cohort of study CL-001, stratified by dose. Time is shown relative to the first dose.

## **Figure S9.** Individual observed concentration-time profiles of sorfequiline and M3 over the full sampling period following 100 mg tablet administration in the relative bioavailability (RBA) cohort of study CL-001, stratified by food status.

# 2. **Visual Predictive Checks (VPCs)**

**Figure S10.** Visual predictive check of sorfequiline and M3 concentrations versus time after dose for the SAD part of the CL-001 study, stratified by dose level, food status and analyte. Dots represent observed concentrations; the solid red line shows the observed median, and the red shaded area represents the 95% confidence interval.

**Figure S11.** Visual predictive check of sorfequiline and M3 concentrations versus time after dose for the MAD part of the CL-001 study, stratified by dose and analyte; all participants were fed. Dots represent observed concentrations; the solid red line shows the observed median, and the red shaded area represents the 95% confidence interval. * For data collected between day 1 and day 14, time after dose reflects time since the first dose.

**Figure S12.** Visual predictive check of sorfequiline and M3 concentrations versus time after dose for the RBA part of the CL-001 study, stratified by food status and analyte; all received 100 mg. Dots represent observed concentrations; the solid red line shows the observed median, and the red shaded area represents the 95% confidence interval.

**Figure S13.** Visual predictive check of sorfequiline and M3 concentrations versus time after dose for the CL-002 study, stratified by analyte. Dots represent observed concentrations; the solid red line shows the observed median, and the red shaded area represents the 95% confidence interval.

**Figure S14.** Prediction-corrected visual predictive check of sorfequiline and M3 concentrations versus time since first dose for the single-dose data (SAD and RBA cohorts), stratified by analyte, up to 360 hours after dosing. The solid red line represents the median of the prediction-corrected observed concentrations, and the red shaded area represents the simulation-based 95% confidence interval for the median. The 10th and 90th percentiles of the prediction-corrected observed concentrations are shown by dashed red lines, and the 95% confidence intervals for the corresponding model-predicted percentiles are shown by blue shaded areas. Blue circles represent observed concentrations after prediction correction.

**Figure S15.** Prediction-corrected visual predictive check of sorfequiline and M3 concentrations versus time since first dose for the multiple-dose data (MAD and CL-002 cohorts), stratified by analyte, up to 360 hours after dosing. The solid red line represents the median of the prediction-corrected observed concentrations, and the red shaded area represents the simulation-based 95% confidence interval for the median. The 10th and 90th percentiles of the prediction-corrected observed concentrations are shown by dashed red lines, and the 95% confidence intervals for the corresponding model-predicted percentiles are shown by blue shaded areas. Blue circles represent observed concentrations after prediction correction.

**Figure S16.** Prediction-corrected visual predictive check of sorfequiline and M3 concentrations versus time since first dose for the single-dose data (SAD and RBA cohorts), stratified by analyte. The solid red line represents the median of the prediction-corrected observed concentrations, and the red shaded area represents the simulation-based 95% confidence interval for the median. The 10th and 90th percentiles of the prediction-corrected observed concentrations are shown by dashed red lines, and the 95% confidence intervals for the corresponding model-predicted percentiles are shown by blue shaded areas. Blue circles represent observed concentrations after prediction correction.

**Figure S17.** Prediction-corrected visual predictive check of sorfequiline and M3 concentrations versus time since first dose for the multiple-dose data (MAD and CL-002 cohorts), stratified by analyte. The solid red line represents the median of the prediction-corrected observed concentrations, and the red shaded area represents the simulation-based 95% confidence interval for the median. The 10th and 90th percentiles of the prediction-corrected observed concentrations are shown by dashed red lines, and the 95% confidence intervals for the corresponding model-predicted percentiles are shown by blue shaded areas. Blue circles represent observed concentrations after prediction correction.

# 3. **S1. NONMEM control stream for the final model**

```
; Settings for the memory of NONMEM
$SIZES      PD=-1000 LVR=-150 LTH=-200 MAXFCN=10000000  LNP4=-150000
;----------------------------------------------------------------------------------------------------------------$PROBLEM    -
;----------------------------------------------------------------------------------------------------------------$INPUT   -
;--------------------------------------------------------------------------------------------------------------$DATA  -
;---------------------------------------------------------------------------------------------------------------- $ABB DERIV2=NO ; Prevents  the computation of second derivatives, which are needed only for the Laplacian method.
;----------------------------------------------------------------------------------------------------------------$SUBROUTINE ADVAN5 TRANS1
;----------------------------------------------------------------------------------------------------------------$MODEL
NCOMPARTMENTS = 10
COMP=(TRANSIT1, DEFDOSE)  ; 1 GUT TRANSIT 1
COMP=(TRANSIT2) ; 2 GUT TRANSIT 2
COMP=(TRANSIT3) ; 3 GUT TRANSIT 3
COMP=(ABS)   ; ABS CMT
COMP=(CENTRAL DEFOBSERVATION)  ; CENTRAL CMT - PARENT
COMP=(PERI1)  ; PERIPH 1 CMT - PARENT
COMP=(PERI2)  ; PERIPH 2 CMT - PARENT
COMP=(M3_Central)  ; CENTRAL CMT - METABOLITE
COMP=(M3_PERI1)  ; PERIPH 1 CMT - METABOLITE
COMP=(M3_PERI2)  ; PERIPH 2 CMT - METABOLITE
;----------------------------------------------------------------------------------------------------------------$PK
;-------------------------BETWEEN OCCASION VARIABILITY-------------------------------------
BOVCL  = 0
BOVBIO = 0
BOVKA  = 0
BOVMTT = 0

IF(OCC==1) THEN
 BOVCL = ETA(17)
 BOVBIO = ETA(20)
 BOVKA = ETA(23)
 BOVMTT = ETA(26)
ENDIF

IF (OCC==2) THEN
 BOVCL = ETA(18)
 BOVBIO = ETA(21)
 BOVKA = ETA(24)
 BOVMTT = ETA(27)
ENDIF

IF(OCC==3) THEN
 BOVCL = ETA(19)
 BOVBIO = ETA(22)
 BOVKA = ETA(25)
 BOVMTT = ETA(28)
ENDIF
;-------------------------BETWEEN SUBJECT VARIABILITY----------------------------------------
BSVCL   = ETA(1)
BSVCL_METABOLITE = ETA(2)
BSVQ    = ETA(3)
BSVV2   = ETA(4)
BSVQ2   = ETA(5)
BSVV3   = ETA(6)
BSVKA   = ETA(7)
BSVBIO  = ETA(8)
BSVMTT  = ETA(9)
BSVV4   = ETA(10)
BSVV    = ETA(11)
BSVQ3   = ETA(12)
BSVV5   = ETA(13)
BSVQ4   = ETA(14)
BSVV6   = ETA(15)
BSVFAM   = ETA(16)
;-------------------------COVARIATES IMPLEMENTATION-----------------------------------------
; ------- Typical value of covariate used as body size descriptors
TVFFM = 53   ; typical value of fat-free mass
;--------- Allometric scaling (using fat-free mass)------------------
ALLMCL_FFM = (FFM/TVFFM)**0.75
ALLMV_FFM = (FFM/TVFFM)
;-------------------------Covariate Implementation-------------------------------------------------------
;---------Implementing effect of food on KA------------------------------------------------------------
FOOD_KA = 1
IF (FED.EQ.1) FOOD_KA = 1 + THETA(21)
;---------Implementing effect of food on MTT of the tablet formulation----------------------------
FOOD_MTT = 1
IF (SOLUTION.EQ.0.AND.FED.EQ.1) FOOD_MTT = 1 + THETA(22)
;-----------Implementing effect on BIO for CL-002 study ---------------------------------
BIO_PART4 = 1
IF (PART.EQ.4) BIO_PART4 = 1 + THETA(25)
;-----------Implementing effect on BIO for 400 and 800mg ------------------------------------------
HIGH_DOSE_BIO = 1
IF (DOSES.EQ.400.OR.DOSES.EQ.800) HIGH_DOSE_BIO = 1 + THETA(26)
;---------Implementing effect of food on FAM----------------------------------------------------------
IF (FED.EQ.0) TVFAM = THETA(23)
IF (FED.EQ.1) TVFAM = THETA(24)
;---------Typical values of parameters--------------------------------------------------------------------
TVCL = THETA(1)*ALLMCL_FFM
TVV = THETA(2)*ALLMV_FFM
TVQ = THETA(3)*ALLMCL_FFM
TVV2 = THETA(4)*ALLMV_FFM
TVQ2 = THETA(5)*ALLMCL_FFM
TVV3 = THETA(6)*ALLMV_FFM
TVKA = THETA(7)*FOOD_KA
TVBIO = THETA(8)*BIO_PART4*HIGH_DOSE_BIO
TVMTT = THETA(9)*FOOD_MTT
TVNN = THETA(10)
TVV4 = THETA(13)*ALLMV_FFM
TVCL_METABOLITE = THETA(14)*ALLMCL_FFM
TVV5 = THETA(15)*ALLMV_FFM
TVQ3 = THETA(16)*ALLMCL_FFM
TVV6 = THETA(17)*ALLMV_FFM
TVQ4 = THETA(18)*ALLMCL_FFM

RHO = THETA(27) ; Correlation between parent and metabolite residual errors
;-----Define parameters ----------------------------------------------------------------------------
CL  = TVCL*EXP(BSVCL + BOVCL)
V   = TVV*EXP(BSVV)
Q   = TVQ*EXP(BSVQ)
V2  = TVV2*EXP(BSVV2)
Q2  = TVQ2*EXP(BSVQ2)
V3  = TVV3*EXP(BSVV3)
KA  = TVKA*EXP(BSVKA + BOVKA)
BIO = TVBIO*EXP(BSVBIO + BOVBIO)
MTT = TVMTT*EXP(BSVMTT + BOVMTT)
NN  = TVNN
V4  = TVV4*EXP(BSVV4)
CL_METABOLITE = TVCL_METABOLITE*EXP(BSVCL_METABOLITE)
V5  = TVV5*EXP(BSVV5)
Q3  = TVQ3*EXP(BSVQ3)
V6  = TVV6*EXP(BSVV6)
Q4  = TVQ4*EXP(BSVQ4)

FAM_TV_LOGIT = LOG(TVFAM /(1-TVFAM)); FAM (fraction of dose absorbed as metabolite) in the logit space
FAM_LOGIT = FAM_TV_LOGIT +(BSVFAM) ; Variability in the logit space
FAM = 1/(1+EXP(-FAM_LOGIT)); back to normal space - This is the actual FAM
;-----Re-parameterization----------------------------------------------------------------------------------
F1  = BIO/(657.55*1000)    ; molar conversion
S5 = V
KTR = (NN+1)/MTT
K12 = KTR
K23 = KTR
K34 = KTR
K45 = KA*(1-FAM)
K48 = KA*FAM
K56 = Q/V
K65 = Q/V2
K57 = Q2/V
K75 = Q2/V3
K58 = CL/V
K89 = Q3/V4
K98 = Q3/V5
K8T10 = Q4/V4
K10T8 = Q4/V6
K80 = CL_METABOLITE/V4

;----------------------------------------------------------------------------------------------------------------
; Initial amounts
A_0(1)  = 1E-12 ; Transit 1
A_0(2)  = 1E-12 ; Transit 2
A_0(3)  = 1E-12 ; Transit 3
A_0(4)  = 1E-12 ; Absorption compartment
A_0(5)  = 1E-12 ; Parent central compartment
A_0(6)  = 1E-12 ; Parent peripheral compartment 1
A_0(7)  = 1E-12 ; Parent peripheral compartment 2
A_0(8)  = 1E-12 ; Metabolite central compartment
A_0(9)  = 1E-12 ; Metabolite peripheral compartment 1
A_0(10) = 1E-12 ; Metabolite peripheral compartment 2
;-------------Residual variability---------------------------------------------------------------------------
$ERROR
LLOQ_P = 0.001 ; mg/L
LLOQ_M = 0.001 ; mg/L

;--------------Parent------------------------------------------------------------------------------------------
CENS_THR_P = LLOQ_P
CP = (A(5)/V)*(657.55*1000)

IPRED_P = CP
PROP_P = IPRED_P*THETA(11)
ADD_P = THETA(12)+(CENS_THR_P*0.2)

 IF (ICALL/=4.AND.BLQ==1.AND.DVID==1) THEN
 ADD_P = ADD_P +(LLOQ_P*1) ; All BLQ observations were imputed as zero and were assigned additional uncertainty (100% LLOQ).
ENDIF
W_P= SQRT((ADD_P)**2+(PROP_P)**2)
;--------------Metabolite------------------------------------------------------------------------------------- CM = (A(8)/V4)*(643.55*1000)
IPRED_M = CM
CENS_THR_M = LLOQ_M

PROP_M = IPRED_M*THETA(19)
ADD_M = THETA(20)+(CENS_THR_M*0.2)

IF (ICALL/=4.AND.BLQ==1.AND.DVID==2) THEN
 ADD_M = ADD_M +(CENS_THR_M*1) ; All BLQ observations were imputed as zero and were assigned additional uncertainty (100% LLOQ).
ENDIF
W_M = SQRT(ADD_M**2+PROP_M**2)
; Correlated parent-metabolite residual error
ERROR_P  = W_P * ERR(1)
ERROR_M  = W_M *(RHO*ERR(1)+SQRT(1-RHO**2) * ERR(2))

;Redefine IPRED & weighting----------------------------------------------------------------------------
IPRED = IPRED_P
W     = W_P
ERROR_TERM = ERROR_P

;METABOLITE
IF (DVID==2) THEN
IPRED = IPRED_M
W     = W_M
ERROR_TERM = ERROR_M
ENDIF

; Protective code---------------------------------------------------------------------------------------------
IF (W.LE.0.000001) W=0.000001
IRES=DV-IPRED
IWRES=IRES/W
Y = IPRED + ERROR_TERM

; To prevent simulation (ICALL==4) of negative values. It set a positive lower bound for Y, so that VPCs in the log-scale can be plotted
IF (DVID==1.AND.ICALL==4.AND.Y<=CENS_THR_P) Y = 0.0001
IF (DVID==2.AND.ICALL==4.AND.Y<=CENS_THR_M) Y = 0.0001

; To calculate time after dose.
IF(AMT>0) THEN
 TIMEDOSE = TIME
 AMOUNTDOSE = AMT
ENDIF

TAD = TIME-TIMEDOSE
TSOD = TAD
;------------------------------------------RETRIEVE AMOUNT IN EACH COMPARTMENT------
AA1 = A(1)
AA2 = A(2)
AA3 = A(3)
AA4 = A(4)
AA5 = A(5)
AA6 = A(6)
AA7 = A(7)
AA8 = A(8)
AA9 = A(9)
AA10 = A(10)
;----------------------------------------------------------------------------------------------------------------
$THETA
(0, 4.24,50) ; 1 CL (L/h)
(0, 17.1,800) ; 2 V (L)
(0, 6.64,30) ; 3 Q (L/h)
(0, 214,800) ; 4 V2 (L)
(0, 10.4,30) ; 5 Q2 (L/h)
(0, 9180,35000) ; 6 V3 (L)
(0, 0.127,20) ; 7 KA (1/h)
(1) FIX ; 8 BIO
(0, 1.13,5) ; 9 MTT (h)
(0, 2,20) FIX ; 10 NN (.)
(0, 0.217,1) ; 11 Prop (%)
(0) FIX ; 12 Add (mg/L)
(0, 3330,35000) ; 13 V4 (L)
(0, 38.4,500) ; 14 CL_METABOLITE (L/h)
(0, 6460,35000) ; 15 V5 (L)
(0, 43.5,200) ; 16 Q3 (L/h)
(0, 2870,35000) ; 17 V6 (L)
(0, 192,500) ; 18 Q4 (L/h)
(0, 0.165,1) ; 19 PROP_M (%)
(0) FIX ; 20 ADD_M (mg/L)
(-0.99, 0.136,5) ; 21 FOOD_KA (%)
(-0.99, 1.88,5) ; 22 FOOD_MTT
(0, 0.464,1) ; 23 FAM
(0, 0.157,1) ; 24 FAM_FOOD
(-0.99, 0.472,2) ; 25 BIO_PART4 (%)
(-0.99, -0.467,3) ; 26 HIGH_DOSE_BIO (%)
(-0.99, 0.786,1) ; 27 RHO

$OMEGA  BLOCK(2)
 0.289  ;        1 BSV CL
 0.147  0.147  ;  [Eta vs Eta correlation] 2 BSVCL_METABOLITE
$OMEGA  BLOCK(1)
 0 FIX  ;   3 BSV Q
$OMEGA  BLOCK(1)
 0 FIX  ;   4 BSV V2
$OMEGA  BLOCK(1)
 0 FIX  ;   5 BSV Q2
$OMEGA  BLOCK(1)
 0 FIX  ;   6 BSV V3
$OMEGA  BLOCK(1)
 0 FIX  ;   7 BSV KA
$OMEGA  BLOCK(1)
 0 FIX  ;   8 BSV BIO
$OMEGA  BLOCK(1)
 0 FIX  ;   9 BSV MTT
$OMEGA  BLOCK(1)
 0 FIX  ;  10 BSVV4
$OMEGA  BLOCK(1)
 0 FIX  ;   11 BSV V
$OMEGA  BLOCK(1)
 0 FIX  ;   12 BSV Q3
$OMEGA  BLOCK(1)
 0 FIX  ;   13 BSV V5
 $OMEGA  BLOCK(1)
 0 FIX  ;   14 BSV Q4
$OMEGA  BLOCK(1)
 0 FIX  ;   15 BSV V6
$OMEGA  BLOCK(1)
 0.429 ;   16 BSV FAM
;----------------------------------------------------------------------------------------------------------------
$OMEGA  BLOCK(1)
 0 FIX  ;   17 BOVCL
$OMEGA  BLOCK(1)
 0 FIX
$OMEGA  BLOCK(1)
 0 FIX
;--------------------------------------------------------------------------------------------------------------
$OMEGA  BLOCK(1)
 0.282 ;   20 BOVBIO
$OMEGA  BLOCK(1) SAME
$OMEGA  BLOCK(1) SAME
;----------------------------------------------------------------------------------------------------------------
$OMEGA  BLOCK(1)
 0.069 ;   23 BOVKA
$OMEGA  BLOCK(1) SAME
$OMEGA  BLOCK(1) SAME
;----------------------------------------------------------------------------------------------------------------
$OMEGA  BLOCK(1)
 0.236 ;   26 BOVMTT
$OMEGA  BLOCK(1) SAME
$OMEGA  BLOCK(1) SAME
;----------------------------------------------------------------------------------------------------------------
$SIGMA 1 FIX
$SIGMA 1 FIX
;----------------------------------------------------------------------------------------------------------------
$ESTIMATION MSFO=run325.msf MAXEVAL=9999 PRINT=1 METHOD=1 INTER NOABORT
NONINFETA=1 ETASTYPE=1 ; REPEAT ;
$COVARIANCE PRINT=E ; MATRIX=S
;----------------------------------------------------------------------------------------------------------------
$TABLE -
```
